# Identification of the Third Binding Site of Arsenic in *Human* Arsenic (III) Methyltransferase

**DOI:** 10.1371/journal.pone.0084231

**Published:** 2013-12-31

**Authors:** Xiangli Li, Zhirong Geng, Jiayin Chang, Shuping Wang, Xiaoli Song, Xin Hu, Zhilin Wang

**Affiliations:** 1 State Key Laboratory of Coordination Chemistry, School of Chemistry and Chemical Engineering, Nanjing University, Nanjing, PR China; 2 School of Chemistry and Chemical Engineering, Yangzhou University, Yangzhou, PR China; 3 Modern Analysis Center of Nanjing University, Nanjing, PR China; Centro Nacional de Biotecnologia – CSIC, Spain

## Abstract

Arsenic (III) methyltransferase (AS3MT) catalyzes the process of arsenic methylation. Each arsenite (iAs^3+^) binds to three cysteine residues, methylarsenite (MMA^3+^) binds to two, and dimethylarsenite (DMA^3+^) binds to one. However, only two As-binding sites (Cys156 and Cys206) have been confirmed on *human* AS3MT (hAS3MT). The third As-binding site is still undefined. Residue Cys72 in *Cyanidioschyzon merolae* arsenite S-adenosylmethyltransferase (CmArsM) may be the third As-binding site. The corresponding residue in hAS3MT is Cys61. Functions of Cys32, Cys61, and Cys85 in hAS3MT are unclear though Cys32, Cys61, and Cys85 in *rat* AS3MT have no effect on the enzyme activity. This is why the functions of Cys32, Cys61, and Cys85 in hAS3MT merit investigation. Here, three mutants were designed, C32S, C61S, and C85S. Their catalytic activities and conformations were determined, and the catalytic capacities of C156S and C206S were studied. Unlike C85S, mutants C32S and C61S were completely inactive in the methylation of iAs^3+^ and active in the methylation of MMA^3+^. The catalytic activity of C85S was also less pronounced than that of WT-hAS3MT. All these findings suggest that Cys32 and Cys61 markedly influence the catalytic activity of hAS3MT. Cys32 and Cys61 are necessary to the first step of methylation but not to the second. Cys156 and Cys206 are required for both the first and second steps of methylation. The S^C32^ is located far from arsenic in the WT-hAS3MT-SAM-As model. The distances between S^C61^ and arsenic in WT-hAS3MT-As and WT-hAS3MT-SAM-As models are 7.5 Å and 4.1 Å, respectively. This indicates that SAM-binding to hAS3MT shortens the distance between S^C61^ and arsenic and promotes As-binding to hAS3MT. This is consistent with the fact that SAM is the first substrate to bind to hAS3MT and iAs is the second. Model of WT-hAS3MT-SAM-As and the experimental results indicate that Cys61 is the third As-binding site.

## Introduction

Arsenic is a potent toxicant, carcinogen and a therapeutic agent for the treatment of cancer. All three of these effects are closely related to arsenic metabolism [1]–[3]. Arsenic methylation is the main process by which inorganic arsenic (iAs) is metabolized [4]. Arsenic (III) methyltransferase (AS3MT) catalyzes the transfer of methyl groups from S-adenosylmethionine (SAM) to the arsenic (As) atom [5], [6]. Arsenic in the trivalent oxidation state, which has a high affinity to the –SH found on Cys, is believed to bind to AS3MT by forming As-S bonds with the Cys residues of AS3MT [7], [8]. Each iAs^3+^ can bind to three cysteine residues, methylarsenite (MMA^3+^) can bind to two, and dimethylarsenite (DMA^3+^) can bind to one. Each metallothionein molecule has twenty Cys residues, so it can bind to up to six iAs^3+^, ten MMA^3+^, or twenty DMA^3+^ molecules, respectively. This is consistent with the coordination chemistry of these arsenicals [9]. The mechanism of arsenic methylation proposed by Hayakawa states that the enzymatic substrates are As-GSH compounds. This means that each iAs^3+^ can bind to three glutathione (GSH) molecules, MMA^3+^ to two, and DMA^3+^ to one [10]–. The mechanism of arsenic methylation proposed by Naranmandura also shows that iAs^3+^ binds to protein via the formation of three As-S bonds [13]. Both these mechanisms suggest that the binding of iAs^3+^ to three Cys residues in a single enzyme is possible.

Cys residues are highly important to enzymes in several ways. They help maintain enzyme structure and regulate enzyme activity [14]. In bacteria, Cys residues have been found to be involved in the reduction of arsenate to arsenite [15]. Cys residues in AS3MT play essential roles in the structure and function of protein [7], [16]–[19]. The functions of some AS3MT Cys residues have been studied in different species. Cys157 and Cys207 in *mouse* AS3MT and Cys156 in *rat* AS3MT have been shown to be sites of As binding and enzymatic activity [16], [17]. There are 14 cysteine residues (Cys32, Cys61, Cys72, Cys85, Cys156, Cys206, Cys226, Cys250, Cys271, Cys334, Cys360, Cys368, Cys369, and Cys375) in *human* AS3MT (hAS3MT) [5], [20]. There are four fully conserved residues Cys32, Cys61, Cys156, and Cys206 in hAS3MT. Their locations were deduced using the sequence alignment of the AS3MTs of various species. In hAS3MT, Cys156 and Cys206 are believed to be the As-binding sites [18]. The functions of other Cys residues in hAS3MT, such as Cys72, Cys226, Cys250, Cys271, Cys334, Cys360, and Cys375, have also been studied [18], [19]. The hAS3MT mutants C72S and C250S, which have destructive conformations because C72S and C250S almost completely lack β-pleated sheets, are both completely inactive in the methylation of iAs^3+^, which indicates that Cys72 and Cys250 are essential to maintenance of the conformation of hAS3MT [18], [19]. The adjacent hAS3MT residues Cys368 and Cys369 may form disulfide bond [17], as has been observed in adjacent cysteine residues in von Willebrand factor (VWF) and nicotinic acetylcholine receptors (nAChRs) [21]. The *rat* AS3MT residues Cys32, Cys61, and Cys85 cannot affect the catalytic activity of the enzyme [17]. However, the functions of residues Cys32, Cys61, and Cys85, which are located in the N-terminal region of hAS3MT, have not been investigated.

Residue Cys72 in *Cyanidioschyzon merolae* arsenite S-adenosylmethyltransferase (CmArsM) might be the third As-binding site [7], [22]. The corresponding residue in hAS3MT is Cys61. Only two As-binding sites, Cys156 and Cys206, have been found in hAS3MT [18]. No third As-binding site has been confirmed in hAS3MT. Finding out the third As-binding site in hAS3MT would facilitate investigations of the mechanism by which iAs^3+^ binds to hAS3MT and the mechanism underlying arsenic methylation. In the present paper, the functions of residues Cys32, Cys61, and Cys85 were evaluated in hAS3MT. Here, residues Cys32, Cys61, and Cys85 were replaced by Ser, and mutants C32S, C61S, and C85S were obtained by site-directed mutagenesis. Although in previous studies, the mutants C156S and C206S were inactive in the methylation of iAs^3+^ and GSH served as the reductant, no previous work has determined whether they can catalyze the methylation of MMA^3+^ [18]. For this reason, the catalytic activity of C156S and C206S with respect to the methylation of iAs^3+^ and MMA^3+^ was further studied in different reductant systems. The WT-hAS3MT-SAM, WT-hAS3MT-As, and WT-hAS3MT-SAM-As models were built. The experimental results and model of WT-hAS3MT-SAM-As suggest that Cys61 is the third binding site of iAs^3+^.

## Materials and Methods

### Caution

Arsenical compounds are known human carcinogens and should be handled accordingly [23].

### Materials

The expression host, *Escherichia coli* BL21 (DE3) pLysS, was purchased from Novagen. The restriction enzymes, dNTPs and PrimerSTAR HS DNA polymerase, were obtained from Takara. The wild-type hAS3MT expression plasmid, pET-32a-hAS3MT, was derived from an earlier study [20]. SAM, glutathione (GSH), tris (2-carboxyethyl) phosphine hydrochloride (TCEP), dihydrolipoic acid (DHLA), isopropyl β-D-thiogalactopyranoside (IPTG), and bovine serum albumin (BSA) were purchased from Sigma. The pH 7.0 phosphate-buffered saline (PBS) buffer was prepared by mixing appropriate volumes of Na_2_HPO_4_ and NaH_2_PO_4_ into a 25 mM stock solution. Arsenicals were purchased from J&K Chemical Ltd. MMA^3+^ was obtained by reducing pentavalent monomethylarsenic (MMA^5+^) using L-cysteine at 90°C for 1 h [8], [24].

### Multiple sequence alignment of various species AS3MT

Multiple sequence alignment of AS3MT between various species was performed using Clustal W (http://www.genome.jp/tools/clustalw/) and analyzed using the BoxShade server (http://www.ch.embnet.org/software/BOX_form.html). The sequences of these proteins were obtained from the National Center for Biotechnical Information (NCBI) database.

### Preparation of hAS3MT mutants

The plasmid pET-32a-*hAS3MT* was subjected to site-directed mutagenesis of *hAS3MT* [18]–[20]. The primers used for site-directed mutagenesis are listed in Table 1. The *hAS3MT* mutants were subjected to DNA sequencing using the double-stranded dideoxy method to ensure that no errors had been introduced during amplification [25]. E. coli BL21 (DE3) pLysS were transformed using vectors carrying different mutations of hAS3MT genes. Single colonies were picked from standard ampicillin-containing agar plates. Protein expression and purification were performed in accordance with previously described protocols [20]. All proteins were dialyzed against PBS (25 mM, pH 7.0) at 4°C to remove imidazole and excess salts. Protein concentrations were determined using the method described by Bradford based on a BSA standard curve [26]. The purified proteins were analyzed by sodium dodecyl sulfate polyacrylamide gel electrophoresis (SDS-PAGE) to ensure protein purity and confirm expression.

**Table 1:** Primers used for site-directed mutagenesis.

	Primer sequence
C32S	− 5′-GTGGTCAC**AGA**ACCGTTGG-3′
C61S	− 5′-CCAGACC**GGA**GCCATAATATCTTAGGG-3′
C85S	+ 5′-GTGGTAGAGAT**TCC**TATGTACTTAGCC-3′− 5′-GGCTAAGTACATA**GGA**ATCTCTACCAC-3′
Whole	+ 5′-CGGGATATCATGGCTGCACTTCGTGAC-3′− 5′-CGGGTCGACTTAGTGATGGTGATG-3′

The “Whole” in Table 1 refers upstream and downstream primers for full length gene of the *hAS3MT*.

### Enzyme activity assays

The arsenic methylation activity of the mutants (C32S, C61S, C85S) was determined in an assay system (100 μl) containing 11 μg enzyme, 7 mM GSH, 1 μM iAs^3+^ and 1 mM SAM in PBS (25 mM, pH 7.0) [18], [19]. To determine the kinetic parameters of iAs^3+^, 0.5–500 μM iAs^3+^ was used while the concentrations of all other components remained fixed. To determine the kinetic parameters of SAM, 0.05–1 mM SAM was used while other components remained fixed. For inactive cysteine mutants and previously designed mutants (C156S and C206S), an assay system (100 μl) containing 11 μg enzyme, 7 mM GSH/1 mM tris (2-carboxyethyl) phosphine hydrochloride (TCEP)/100 μM dihydrolipoic acid (DHLA), 1 μM iAs^3+^/MMA^3+^, and 1 mM SAM in PBS (25 mM, pH 7.0) was used. The reaction mixtures were incubated at 37°C for 2 h and then terminated by addition of H_2_O_2_ to a final concentration of 3% to release the arsenicals from proteins and oxidize all arsenic metabolites to pentavalency [10]. After being filtered through a 0.22 µm pore membrane, 20 μl aliquots of the samples were separated on an anion-exchange column (PRP X-100 250 mm ×4.6 mm i.d., 5 µm, Hamilton) and analyzed using HPLC-ICP-MS (Elan 9000, PerkinElmer) at a flow rate of 1.0 ml/min at room temperature [19], [27]–[29]. The arsenical compounds were eluted with a mobile phase of 15 mM (NH_4_)_2_HPO_4_. The pH of the mobile phase was adjusted to 6.0 with H_3_PO_4_. The concentrations of arsenic species were calculated with the working curves prepared using 5, 10, 25, 50, and 100 μg/L of standard arsenic species. Methylation rates were calculated as mole equivalents of methyl groups transferred from SAM to iAs^3+^ (i.e., 1 nmol CH_3_ per 1 nmol MMA or 2 nmol CH_3_ per 1 nmol DMA) [30]. The rate of the methylation reaction follows the rate of noncompetitive substrate inhibition as shown in equation (2): *V = * [S]*V_max_/(K_M_+[S]*+*[S]^2^ */K_I_)* [18], [31]. Here, *V* is the initial velocity of the reaction (pmol CH_3_ transferred/h/mg protein); [S] is the substrate (iAs^3+^) concentration (μM); *V_max_* is the maximal velocity of the reaction (pmol CH_3_ transferred/h/mg protein); *K_M_* is the Michaelis constant of iAs^3+^ (μM); and *K_I_* is the inhibition constant of iAs^3+^ (μM) [32].

### Circular dichroism (CD) and attenuated total reflection Fourier transform infrared (ATR-FTIR) spectra

Circular dichroism (CD) (190–265 nm) spectra of WT-hAS3MT and hAS3MT mutants were recorded with a JASCO-J810 Spectropolarimeter (Jasco Co., Japan) with a 1 mm cell and 10 mm light length at a scanning rate of 50 nm/min. Each spectrum represents the average of three accumulations recorded per mutant protein solution (2 μM in 25 mM PBS, pH 7.0) and the secondary structure parameters of the mutants were calculated using Jwsse32 software with reference CD-Yang. jwr [33]. Baseline correction was automatically carried out with the PBS (25 mM, pH 7.0) spectrum throughout the entire collection process. Attenuated total reflection Fourier transform infrared (ATR-FTIR) spectra were also used to analyze the secondary structure of the mutants. More details about the ATR-FTIR spectra are given in previous works [18]–[20], [34]–[36].

### Modeling of WT-hAS3MT-As-SAM using modeller9v8

Using the SAM-CmArsM and As-CmArsM structures (PDB code 4FR0) as templates, models of WT-hAS3MT-As and WT-hAS3MT-SAM were built with modeller9v8 [22]. The models of WT-hAS3MT-As and WT-hAS3MT-SAM were superimposed using Accelrys Discovery Studio and a model of WT-hAS3MT-As-SAM was built. The quality of the hAS3MT model was estimated using the QMEAN Server (http://swissmodel.expasy.org/qmean/cgi/index.cgi) [37]. Pymol was used to analyze the hAS3MT models [38], [39].

## Results

### Sequence alignment of various species AS3MT

**Figure 1:**
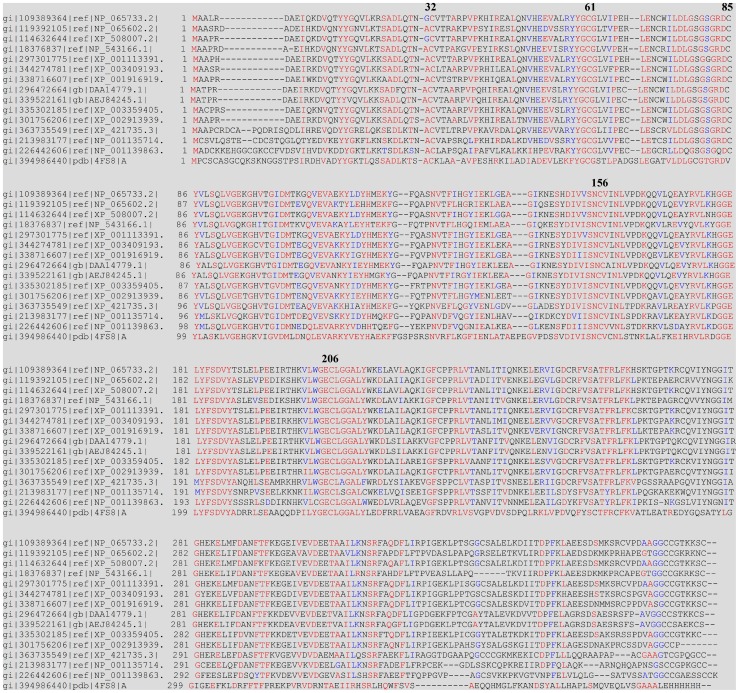
Sequence alignment of AS3MTs from various species. Sequence are denoted by NCBI gi number, which contain NP_065733.2 (*Homo sapiens*), NP_065602.2 (*Mus musculus*), XP_508007.2 (*Pan troglodytes*), NP_543166.1 (*Rattus norvegicus*), XP_001113391.2 *(Macaca mulatta*), XP_003409193.1 (*Loxodonta Africana*), XP_001916919.2 (*Equus caballus*), DAA14779.1 (*Bos taurus*), AEJ84245.1 (*capra hircus*), XP_003359405.1 (*Sus scrofa*), XP_002913939.1 (*Ailuropoda melanoleuca*), XP_421735.3 (*Gallus gallus*), NP_001135714 (*Xenopus (Silurana) tropicalis*), NP_001139863 (*Salmo salar*) and 4FS8 (*Cyanidioschyzon merolae*). The conserved residues are marked in red and the residues Cys32, Cys61, Cys85, Cys156, and Cys206 in hAS3MT are marked with their residue numbers.

Conservation of isolated amino acid residues and short stretches of residues surrounded by variable sequences within a protein often indicates that the conserved element played an important role in that protein's function or structural organization [40]. Multiple sequence alignment of AS3MT was performed across several species using Clustal W software. Results were analyzed using BoxShade server. The sequences of these proteins were obtained from the National Center for Biotechnology Information (NCBI) database. Results showed the cysteine residues Cys32, Cys61, Cys156, and Cys206 in hAS3MT to be absolutely conserved (Figure 1).

### Expression and purification of the hAS3MT mutants

**Figure 2:**
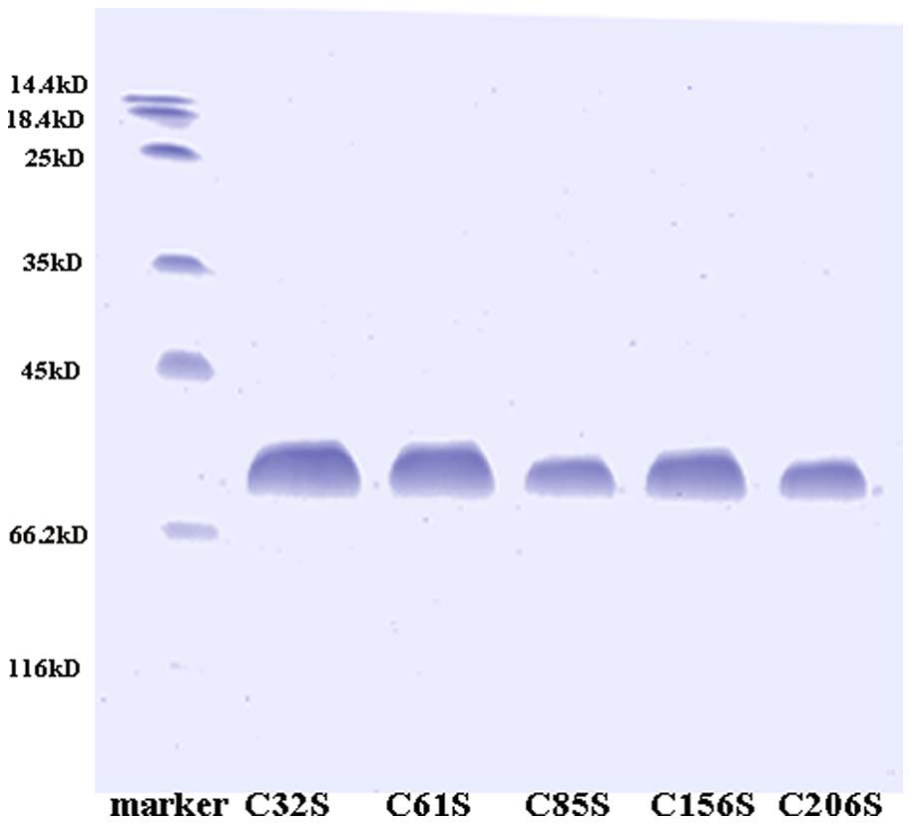
Sodium dodecyl sulfate polyacrylamide gel electrophoresis (SDS-PAGE). 12% SDS-PAGE gel of the purified protein of hAS3MT mutants were stained with Coomassie blue.

The hAS3MT mutants were expressed and purified using a protocol described in previous studies [18]–[20]. All the mutant proteins were expressed successfully. The purity of each mutant protein was confirmed to be over 90% by sodium dodecyl sulfate polyacrylamide gel electrophoresis (SDS-PAGE) (Figure 2). The purity of mutants C156S and C206S, which were designed in a previous work, was also confirmed by SDS-PAGE [18] (Figure 2).

### Catalytic activities of the mutants

**Figure 3:**
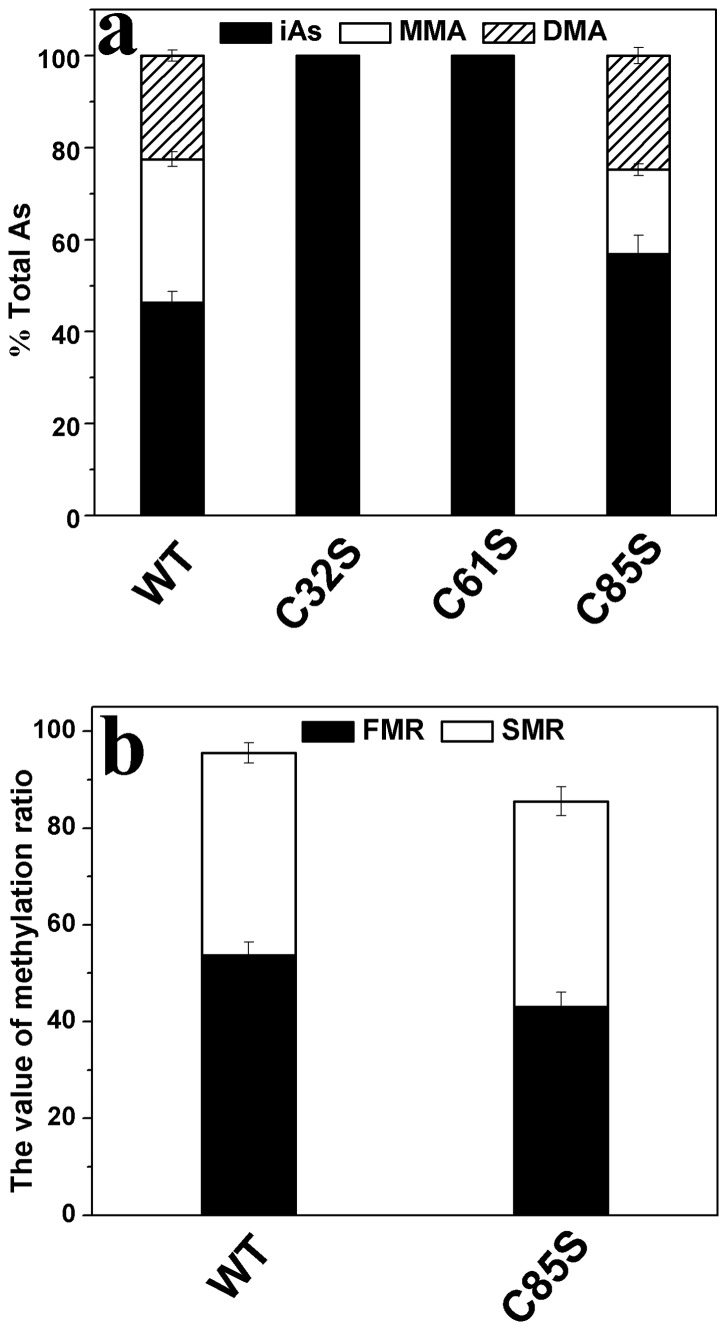
Catalytic capacities of the hAS3MT mutants. Reaction mixtures (100 μl) containing 11 μg enzymes, 1 μM iAs^3+^, 1 mM SAM and 7 mM GSH in PBS (25 mM, pH 7.0) were incubated at 37°C for 2 h and analyzed using HPLC-ICP-MS. The percents of arsenic species (iAs/TAs, MMA/TAs and DMA/TAs) and the two indices (FMR and SMR) of mutant C85S are shown in Figures 3a and 3b. Values are the averages ± S.D. of three independent experiments performed using three independently purified proteins.

The catalytic capacities of the mutants were determined using a reaction system (100 μl) containing 11 μg enzyme, 7 mM GSH, 1 μM iAs^3+^, and 1 mM SAM in PBS (25 mM, pH 7.0). The total arsenic (TAs) concentration was found by adding the concentrations of iAs, MMA, and DMA [39], [41]. According to the pathway of iAs methylation, secondary methylation can only proceed based on first methylation and then parts of the products of this first methylation are later methylated further. To assess the degree of first methylation, not only the primary but also the secondary methylation products must be considered. Two indices, the first methylation ratio (FMR) and the secondary methylation ratio (SMR), were utilized to evaluate methylation capacity. These were calculated as (MMA + DMA)/TAs and DMA/(MMA + DMA), respectively [39], [42]. Using the FMR and SMR to evaluate the arsenic methylation capacity of the mutants has been found to be logical [42]. The relative amount of each arsenic species (iAs/TAs, MMA/TAs and DMA/TAs) and the two indices (FMR and SMR) of the mutants C32S, C61S, and C85S are shown in Figures 3a and 3b, respectively. The mutants C32S and C61S showed no catalytic activity in that there was no methylated arsenic obtained when they were used as the enzymes. iAs/TAs and DMA/TAs of C85S were higher than those of WT, suggesting that the total methylated arsenic (MMA + DMA) reduced when C85S used as the enzyme. The TAs was fixed, so the FMR of C85S decreased when SMR increased compared with those of WT. In general, the methylation capacity of the C85S was lower than that of WT. These results suggest that residues Cys32 and Cys61 affect the catalytic activity of hAS3MT profoundly and that Cys85 influences it slightly.

**Figure 4:**
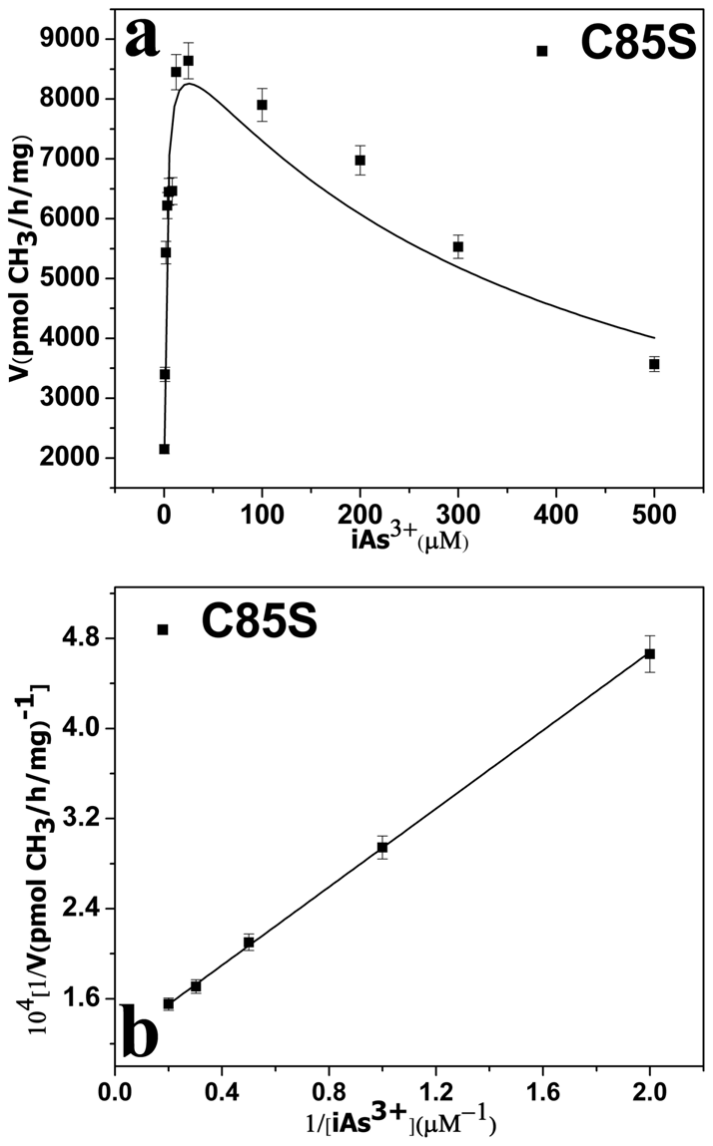
**a Rate of arsenic methylation and Substrate concentration.** The line shows the least squares fit of Eq. (1) to the data. **b Double reciprocal plot of the arsenic methylation rate against the concentration of iAs^3+^.** Reaction mixtures (100 μl) containing 11 μg enzymes, 1 mM SAM, and 7 mM GSH in PBS (25 mM, pH 7.0) were incubated with different concentrations of iAs^3+^ at 37°C for 2 h with H_2_O_2_ treatment before analysis. Values are the averages ± S.D. of three independent experiments performed using three independently purified proteins.

The rate of inhibition of substrate by iAs^3+^ was observed for all active mutants across a wide range of iAs^3+^ concentrations (0.5–500 μM) (Figure 4a). The kinetic parameters of the active mutants are shown in Table 2. They were estimated by fitting Eq. (2) and calculated using a double reciprocal plot (Figure 4b). The two methods produced consistent results. The *K_I_* and *K_M_* values of iAs^3+^ of the mutant C85S were lower than those of WT-hAS3MT (*K_M_*, 3.2 μM; *K_I_*, 0.7 mM; *V_max_*, 19,836 pmol/h/mg [18]). The *V_max_* value of C85S was 45% that of WT-hAS3MT. These results indicate that the affinity of the mutant C85S to iAs is greater than that of WT.

**Table 2:** Kinetic parameters for iAs methylation of the mutant C85S.

	C85S	WT
a ***V***_max_ (pmolCH_3_/mg/h) ×10^3^	9.4±0.6	21.2±1.1
a ***K*** *_M_* (µM)	1.7±0.3	3.2±0.3
a ***K***_I_ (mM)	0.44±0.12	0.76±0.09
b ***K*** *_M_* (µM)	1.5±0.2	3.2±0.7
b ***V***_max_ (pmolCH_3_/mg/h) ×10^3^	8.3±0.6	19.8±1.0
c ***K*** *_M_* (µM)	80.0±7.4	47.8
Relative c ***K*** *_M_*	1.9	1.0

Values represent the average ± S.D. of three independent experiments performed using three independently purified proteins.

^a^ Represents the kinetic parameters of iAs^3+^ estimated from the data in Figure 4a by Eq. (1) using origin 8.0.

^b^ Represents the kinetic parameters of iAs^3+^ calculated from the data in Figure 4b.

^c^ Represents the *K_M_* of SAM.

**Figure 5:**
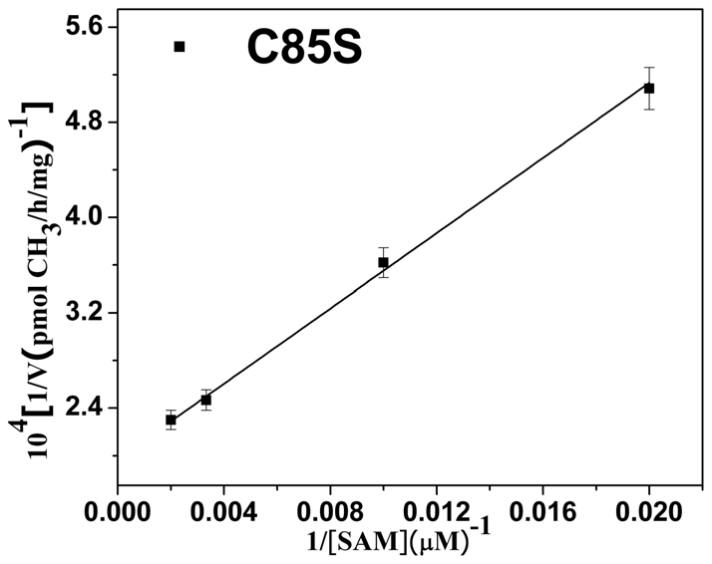
Double reciprocal plot of the arsenic methylation rate versus the concentration of SAM. Reaction mixtures (100 μl) containing 11 μg C85S, 1 μM iAs^3+^, and 7 mM GSH in PBS (25 mM, pH 7.0) were incubated with various concentrations of SAM for 2 h with H_2_O_2_ treatment before analysis. Values are the averages ± S.D. of three independent experiments performed by three independently purified proteins.

The rate of arsenic methylation increased as the concentration of SAM increased. The *K_M_* values of the SAM of the mutants, which reflect the ability of SAM to interact with hAS3MT, were calculated using the double reciprocal plot (Figure 5). They are summarized in Table 2. For the mutant C85S, the *K_M_* values of SAM increased to 80.0 μM, which was slightly higher than that of WT (WT: 47.84 μM [18]). The data show that residue Cys85 has a little effect on the binding of SAM. This is because Cys85 is adjacent to motif I (74-IDLGSGSG-82), which is involved in SAM binding [5], [38], [43].

### Catalytic capacity of the mutants C32S, C61S, C156S, and C206S in the methylation of iAs^3+^ and MMA^3+^ with GSH, TCEP, and DHLA as reductants

**Figure 6:**
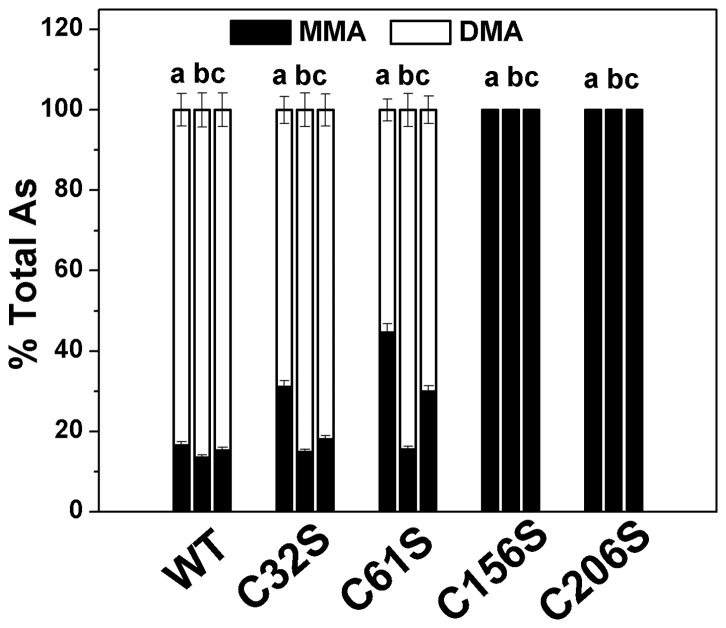
Catalytic capacity of MMA^3+^ for WT-hAS3MT and hAS3MT cysteine mutants. Reaction mixtures (100 μl) containing 11 μg enzymes, 1 μM MMA^3+^, 1 mM SAM, and 7 mM GSH/1 mM TCEP/100 μM DHLA in PBS (25 mM, pH 7.0) were incubated at 37°C for 2 h and analyzed by HPLC-ICP-MS. a, b, and c represent three reductants: 7 mM GSH, 1 mM TCEP, and 100 μM DHLA, respectively. Values are the means ± S.D. of three independent experiments.

To determine the catalytic capacity of the mutants C32S, C61S, C156S, and C206S comprehensively, non-thiol, monothiol and dithiol reductants TCEP, GSH, and DHLA were used in this system and their concentrations were optimized [44], [45]. The cysteine mutants C32S, C61S, C156S, and C206S were all completely inactive in the methylation of iAs^3+^ in the GSH, TCEP, and DHLA systems (data not shown). To determine whether these mutants could catalyze the methylation of MMA^3+^, assay systems (100 μl) containing 11 μg enzyme, 7 mM GSH/1 mM TCEP/100 μM DHLA, 1 μM MMA^3+^ and 1 mM SAM in PBS (25 mM, pH 7.0) were incubated at 37°C for 2 h. The results are shown in Figure 6. The mutants C156S and C206S were completely inactive in MMA^3+^ methylation in the GSH, TCEP, and DHLA systems. C32S and C61S catalyzed the methylation of MMA^3+^ to DMA in all three systems. The catalytic capacities of C32S and C61S were similar to that of WT in the TCEP system, but they were less pronounced than those of WT in the GSH and DHLA systems.

### Conformations of C32S, C61S, and C85S

**Figure 7:**
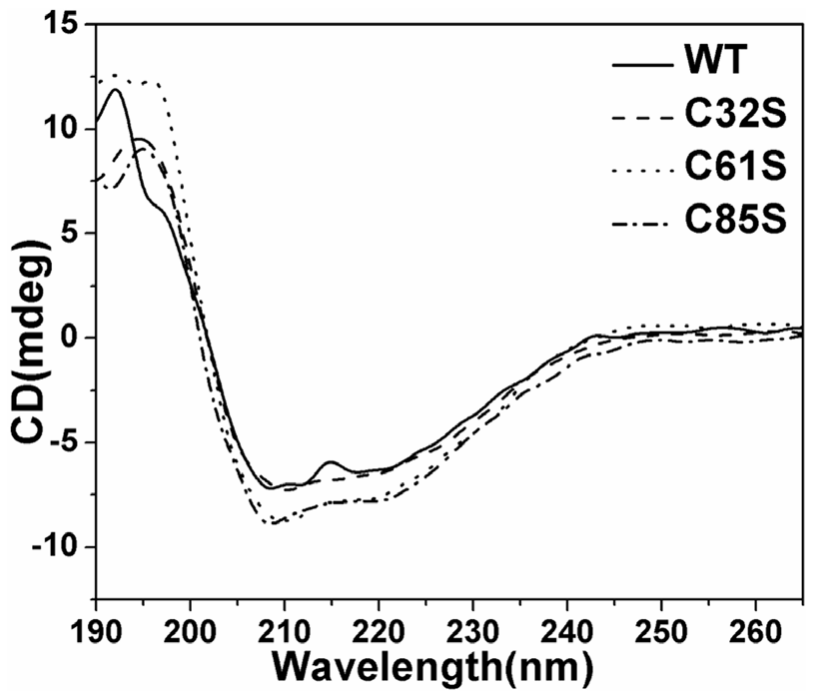
CD spectra of hAS3MT and the mutants. Spectra were taken at protein concentrations of 2 μM at room temperature. Plot is the representative of three independent measurements performed using three independently purified proteins.

The CD spectrum was examined to determine whether the corresponding mutations could cause important conformational changes in the protein [46], [47]. The CD spectra of the three mutants and WT-hAS3MT (Figure 7) showed the intensities of the peaks (208 nm and 220 nm) of mutants C61S and C85S to be higher than those of the wild-type enzyme. These results suggest that the conformations of mutants C61S and C85S are different from those of WT. Secondary structure was computed using Jwsse32 software with reference CD-Yang. jwr (Table 3). WT-hAS3MT has been estimated to be made up of 29.0% α-helixes, 23.9% β-pleated sheets, 17.9% β-turns, and 29.2% random coils [18]. The mutants all have more β-pleated sheets and fewer β-turns and random coils, especially C61S. The content of α-helixes in all mutants except C85S was slightly higher than in WT. The data indicate that the secondary structure of the three mutants, especially C61S, differ from that of WT. The CD spectra of the mutants C156S and C206S have been recorded and analyzed in previous work, so their CD spectra are not been presented in this paper [18].

**Table 3:** Secondary structures of WT-hAS3MT and the mutants estimated using CD spectroscopy.

	α-helix (%)	β-pleated (%)	β-turn (%)	Random (%)
C32S	31.7±1.2	30.4±1.1	14.0±0.7	23.6±0.5
C61S	33.5±1.8	36.9±1.6	8.5±0.4	21.1±0.5
C85S	26.5±1.3	31.9±2.2	14.5±0.5	27.1±0.8
WT	29.0±2.2	23.9±1.9	17.9±1.7	29.2±1.4

Values represent the mean ± S.D. of three independent experiments. The parameters were analyzed using the Jasco secondary structure manager with the reference CD data-Yang. jwr in PBS (25 mM, pH 7.0) at room temperature.

**Figure 8:**
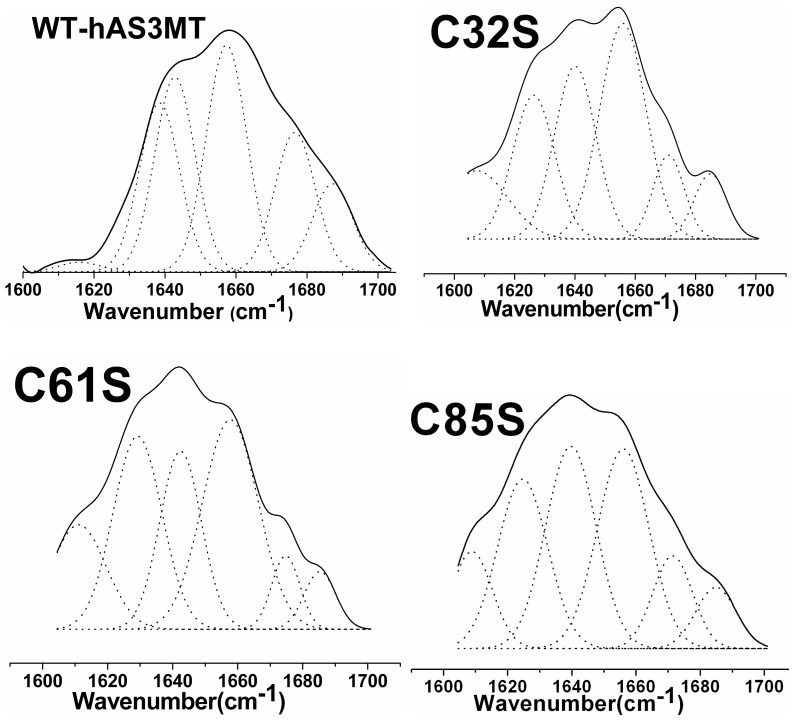
Curve-fitted amide I region of the mutants. The component peaks are the result of curve-fitting using a Gaussian shape. The solid lines represent the experimental FTIR spectra after Savitzky-Golay smoothing, and the dashed lines represent the fitted components. Plot is the representative of three independent measurements carried out using three independently purified proteins.

ATR-FTIR assays were performed and the resulting amide I bands were analyzed to further confirm the secondary structure of the mutants. The original and curve-fitting FTIR spectra of the mutants are shown in Figure 8. There were six component bands in the amide I bands of the mutants. In terms of well-established assignment criteria (1610–1640 cm^−1^: β-pleated sheet, 1640–1650 cm^−1^: random coil, 1650–1658 cm^−1^: α-helix, and 1660–1700 cm^−1^: β-turn), the nature of each secondary structure of the mutants was calculated using the integrated areas of the component bands (Table 4) [37]. The secondary structure of the three mutants obtained using ATR-FTIR was found to be in accordance with those obtained using CD spectra.

**Table 4:** Secondary structures of WT-hAS3MT and the mutants estimated from ATR-FTIR spectroscopy.

	α-helix (%)	β-pleated (%)	β-turn (%)	Random (%)
C32S	32.7±1.5	28.2±0.3	15.5±0.3	23.5±0.8
C61S	31.7±1.6	37.7±2.6	10.6±0.3	20.1±0.6
C85S	27.0±1.2	30.1±2.3	16.1±0.7	26.8±1.1
WT	26.6±3.6	20.7±4.6	24.2±3.2	28.5±4.9

Values represent the mean ± S.D. of three independent experiments. The parameters were analyzed using the origin 7.0.

### Model of WT-hAS3MT-SAM-As

**Figure 9:**
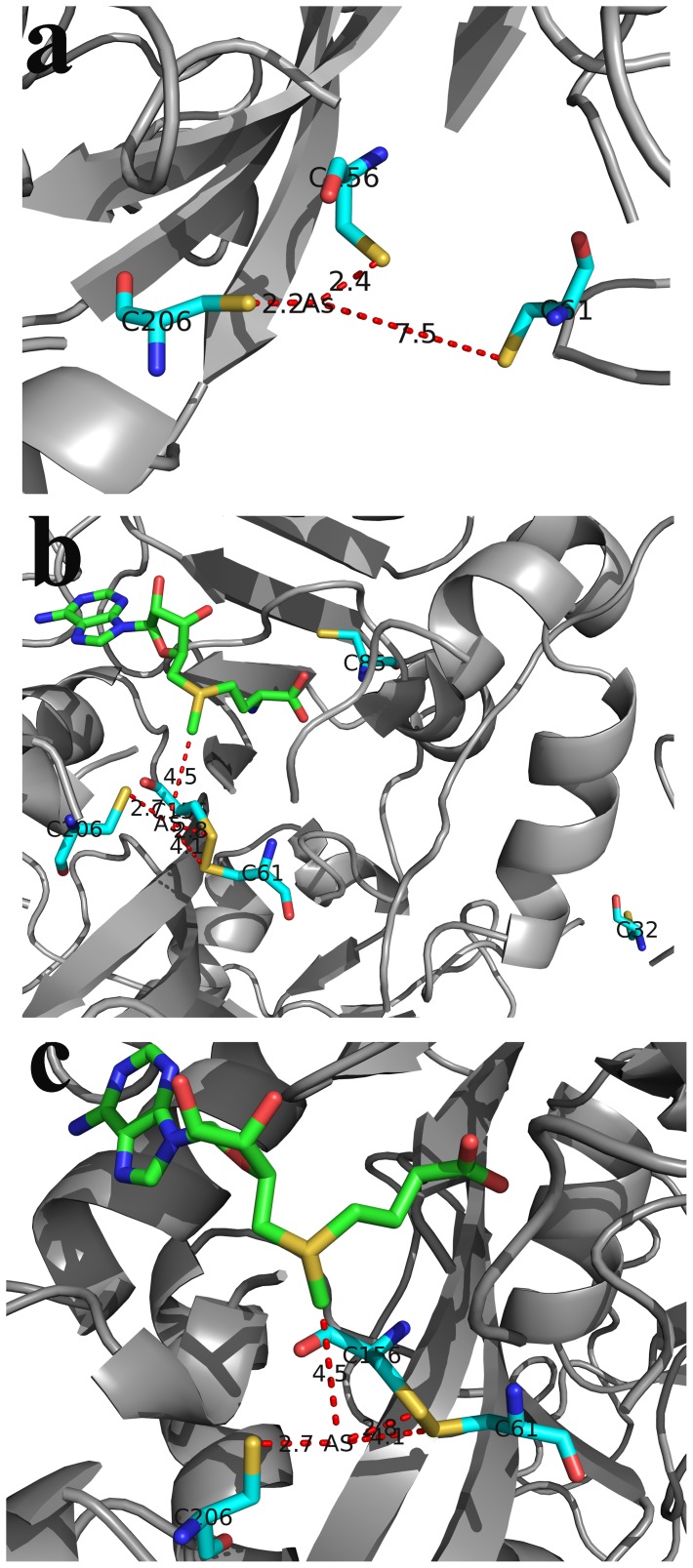
Models of hAS3MT-As and hAS3MT-SAM-As. **a) Model of hAS3MT-As**. The distances between the As atom and S^C61^, S^C156^, S^C206^ are marked. **b) and c) model of hAS3MT-SAM-As.** The distances between the As atom and the CH_3−_S^+^ of SAM, S^C61^, S^C156^, and S^C206^ were determined. **c)** shows an enlargement of **b)**.

Models of WT-hAS3MT-As and WT-hAS3MT-SAM-As are shown in Figures 9a, and 9b, respectively. Figure 9c is an enlargement of WT-hAS3MT-SAM-As. The model of WT-hAS3MT-As shows that the distance between As atom and S^C61^ to be 7.5 Å. The model of WT-hAS3MT-SAM-As shows that the distances between the As atom and S^+^-CH_3_, S^C156^, S^C206^, and S^C61^ were 4.5, 2.8, 2.7, and 4.1 Å, respectively. The distance between S^C61^ and As in the hAS3MT-As model was greater in the hAS3MT-SAM-As model. Figure 9b shows that residues Cys32 and Cys85 are far from the As atom.

## Discussion

The human AS3MT gene is approximately 32-kilobase nucleotide base pairs in size and contains 11 exons. Recently, a number of intronic single-nucleotide polymorphisms (SNPs) have been identified. Only three exonic SNPs, R173W, M287T, and T306I, have been identified in the AS3MT coding region of African-American and Caucasian-American subjects [44], [48]. There are no polymorphisms on the Cys residues of AS3MT in humans. Nevertheless, Cys residues in AS3MT play essential roles in the structure and function of protein [14]. Arsenic in the trivalent oxidation state is believed to bind to AS3MT by forming As-S bonds with the Cys residues of AS3MT [16]–[18]. Cys156 and Cys206 in *human* AS3MT have been shown to be sites of As binding and enzymatic activity [18]. The third binding site of As in hAS3MT is still undefined. Locating the As-binding site in the AS3MT would facilitate understanding of the process by which the As binds to the AS3MT. The SAM-binding domain also has been investigated [39], [43]. Locating As and SAM binding sites in AS3MT helps to calculate the process by which methyl groups are moved from SAM to As using quantum mechanics/molecular mechanics (QM/MM) or other methods of theoretical calculation [49]–[51]. This would promote further understanding of the mechanism of arsenic methylation at a microlevel. In this way, identification the binding sites of As in hAS3MT is meaningful. The methylation process is catalyzed by SAM-dependent methyltransferase and its mechanism is orderly. In various molecules, SAM is the first reactant to bind to SAM-dependent methyltransferases, and the enzymatic substrate is the second [45], [53], [54]. The binding of SAM to the enzyme facilitates the binding of the substrates to the enzyme. Theoretical calculation shows the methyl group transfer process to be a typical in-line S_N_2 nucleophilic substitution reaction in many SAM-dependent methyltransferases [49]–[52]. iAs^3+^ with lone pair can attack the CH_3_ [43]. Arsenic usually binds to the cysteine residues of the AS3MT. As-binding sites have been detected in CmArsM and found to be Cys72, Cys174, and Cys224 [7]. The mutant C72A in CmArSM is inactive in the methylation of iAs^3+^ and active in the methylation of MMA^3+^. Neither C174A nor C224A in CmArsM is active in the methylation of MMA or iAs in CmArsM. Three conserved cysteines are required for the first step of methylation [iAs^3+^ to MMA], but only two (Cys174 and Cys224) are required for the second [MMA^3+^ to DMA]. Note that three conserved residues can provide three sulfur ligands for binding iAs^3+^, but only two sulfur ligands are necessary for the binding of MMA^3+^ [7]. The cysteine mutants C32S and C61S of hAS3MT are inactive in the methylation of iAs^3+^, but they are active in the methylation of MMA^3+^. This indicates that Cys32 and Cys61 are required for the first step of methylation but not for the second. The Cys156 and Cys206 of hAS3MT have been shown to be the As-binding sites in previous study [18]. The corresponding residues in other AS3MTs have also been shown to be As-binding sites and active sites [7], [16], [17]. C156S and C206S are completely inactive in the methylation of iAs^3+^ and MMA^3+^, which indicates that Cys156 and Cys206 are required for the first and second steps of methylation.

The model of WT-hAS3MT-SAM-As shows that the As atom is 2.7 Å, 2.8 Å, 4.1 Å, and 4.5 Å from S^C206^, S^C156^, and S^C61^ of hAS3MT and S^+^-CH_3_ of SAM, respectively. This shows that Cys156 and Cys206 are indeed the binding sites of As. This is consistent with the conclusion drawn in a previous study [18]. Both Cys32 and Cys61 are required for the first step of methylation but not the second. The WT-hAS3MT-SAM-As model shows that Cys32 is much farther away from As than Cys61 is. This suggests that the Cys32 is not the third As-binding site. Cys61 is the third As-binding site. Cys32 probably influences the conformation of hAS3MT. In the model of WT-hAS3MT-As, S^C61^ is 7.5 Å from the arsenic atom. However, in the model of WT-hAS3MT -SAM-As, the Cys61 moves toward the As^3+^-binding site and the S^C61^ moves within 4.1 Å of the As atom. These results suggest that Cys61 participates in iAs^3+^ binding during the first step in the catalytic cycle. The change in the distance between the S^C61^ and arsenic suggests that the SAM first binds to hAS3MT and so promotes the binding of As to hAS3MT. This is consistent with the results of previous studies showing that SAM is the first substrate to bind to hAS3MT. After SAM binding to hAS3MT, the iAs^3+^ with three –OH binds to Cys61, Cys156, and Cys206 in hAS3MT, and then new compound hAS3MT-SAM-As forms, the methyl group could move from the SAM to iAs^3+^. Reductants probably play important roles in reducing the disulfide bond of hAS3MT or binding iAs^3+^, MMA^3+^ and DMA^3+^, which needs further study.
